# Human dosimetry of free ^211^At and *meta*-[^211^At]astatobenzylguanidine (^211^At-MABG) estimated using preclinical biodistribution from normal mice

**DOI:** 10.1186/s40658-020-00326-7

**Published:** 2020-09-22

**Authors:** Naoyuki Ukon, Songji Zhao, Kohshin Washiyama, Noboru Oriuchi, Chengbo Tan, Saki Shimoyama, Miho Aoki, Hitoshi Kubo, Kazuhiro Takahashi, Hiroshi Ito

**Affiliations:** 1 Advanced Clinical Research Center, Fukushima Global Medical Science Center, Fukushima Medical University, 1 Hikariga-oka, Fukushima City, 960-1295 Japan; 2 Preparing Section for School of Health Sciences, Fukushima Medical University, 1 Hikariga-oka, Fukushima City, 960-1295 Japan; 3 Department of Radiology and Nuclear Medicine, Fukushima Medical University, 1 Hikariga-oka, Fukushima City, 960-1295 Japan

**Keywords:** Alpha-emitter, *Meta*-[^211^At]astatobenzylguanidine, Dosimetry, Radionuclide therapy

## Abstract

**Background:**

^211^At is one of the ideal nuclides for targeted radionuclide therapies (TRTs). *Meta*-[^211^At]astatobenzylguanidine (^211^At-MABG) has been proposed for the treatment of pheochromocytoma. To effectively use these radiopharmaceuticals, dosimetry must be performed. It is important to determine the absorbed doses of free ^211^At and ^211^At-MABG to determine the organs that may be at risk when using TRTs. The aim of this study was to estimate human dosimetry from preclinical biodistribution of free ^211^At and ^211^At-MABG in various organs in normal mice.

**Methods:**

Male C57BL/6 N mice were administered 0.13 MBq of free ^211^At or 0.20 MBq of ^211^At-MABG by tail-vein injection. The mice were sacrificed at 5 min, and at 1, 3, 6, and 24 h after the injection (*n* = 5 for each group). The percentage of injected activity per mass in organs and blood (%IA/g) was determined. The human absorbed doses of free ^211^At and ^211^At-MABG were calculated using the Organ Level INternal Dose Assessment/EXponential Modeling (OLINDA/EXM) version 2.0 and IDAC-Dose 2.1.

**Results:**

High uptake of free ^211^At was observed in the lungs, spleen, salivary glands, stomach, and thyroid. The absorbed doses of free ^211^At in the thyroid and several tissues were higher than those of ^211^At-MABG. The absorbed doses of ^211^At-MABG in the adrenal glands, heart wall, and liver were higher than those of free ^211^At.

**Conclusions:**

The absorbed doses of ^211^At-MABG in organs expressing the norepinephrine transporter were higher than those of free ^211^At. In addition, the biodistribution of free ^211^At was different from that of ^211^At-MABG. The absorbed dose of free ^211^At may help predict the organs potentially at risk during TRTs using ^211^At-MABG due to deastatination.

## Background

A new generation of targeted radionuclide therapies (TRTs) involves the use of alpha-particles. One of the TRTs that uses alpha particles is called targeted alpha therapy (TAT). TAT is exclusively cytotoxic and not affected by many of the limitations associated with conventional chemotherapy and radionuclide therapy using electrons. The alpha particles have high-energy deposition [linear energy transfer (LET)] and a limited range in tissue, resulting in strong therapeutic effects with minimal adverse effects on normal organs [1, 2]. Studies on the therapeutic application of alpha-emitters have been carried out using various nuclides [1, 3–9].

Pheochromocytoma originates from the adrenal medulla and sympathetic ganglia, and approximately 10–15% of patients with pheochromocytoma have systemic metastasis, progressing into malignant pheochromocytoma [10, 11]. *Meta*-[^131^I]iodobenzylguanidine (^131^I-MIBG) is a radiopharmaceutical for the systemic treatment of patients with metastatic pheochromocytoma and paraganglioma. ^131^I-MIBG, an analog of guanethidine, concentrates in adrenergic tissue by the same mechanism as that of norepinephrine through the norepinephrine transporter (NET) [12]. Treatment with ^131^I-MIBG has shown limited efficacy even when administered at a high radioactivity such as more than 7.4 GBq [10]. Given the limited treatment approaches currently available for patients with metastatic pheochromocytoma, new effective approaches are being sought out. The alpha-emitting radiopharmaceutical *meta*-[^211^At]astatobenzylguanidine (^211^At-MABG) is an alternative to ^131^I-MIBG for the treatment of malignant pheochromocytoma, because the uptake mechanisms of these radiopharmaceuticals are similar. Astatine is the heaviest element of the halogen group, which also contains iodine; therefore, both astatine and iodine share the same chemical properties. Theoretically, ^211^At-MABG should be more effective and have fewer side effects than ^131^I-MIBG, because alpha-particles have a high LET and a very short range in tissues compared with electrons [1].

Since the decay pathway is 100% alpha-particle emission (5.87 and 7.45 MeV in 42% and 58% of the decays, respectively) during the decay of ^211^At at a half-life of 7.2 h, ^211^At is one of the nuclides available for TAT [13, 14]. However, the biochemical properties of ^211^At and ^211^At-labeled compounds have not been clarified owing to the absence of stable astatine isotopes. In addition, the ^211^At-labeled compounds for TAT can be problematic owing to their rapid deacidification in vivo [15, 16]. Free ^211^At generated by deastatination may accumulate in specific tissues through redistribution. Therefore, it is important to determine the human dosimetry of free ^211^At and ^211^At-labeled compounds in normal tissues to predict the organ potentially at risk when using radiopharmaceuticals for TAT.

Several studies based on planar images of ^211^At obtained using a gamma camera have been reported [17, 18]. However, there are still issues with the spatial resolution of the images and the quantification of the images because of the lack of attenuation correction. Therefore, the absorbed doses in humans were assessed by measuring and extrapolating the in vivo distribution in mice.

Several types of absorbed dose calculation software have been developed for nuclear medicine. The Organ Level INternal Dose Assessment/EXponential Modeling (OLINDA/EXM) versions 1.0, 1.1 (Vanderbilt University, Nashville, TN, USA) and 2.0 (Hermes Medical Solutions, Stockholm, Sweden) were developed as dosimetry software, as reported by Stabin et al. [19, 20]. In addition, IDAC-Dose 2.1 was also created as an internal dosimetry computer program by Anderssen et al. [21].

OLINDA/EXM version 2.0 has realistic human computational phantoms that were based on the International Commission on Radiological Protection (ICRP) Publication 89, i.e., nonuniform rational B-splines voxel-based models. It also includes phantoms for the mouse, rat, and dog. IDAC-Dose 2.1 has a voxel phantom installed describing two adults that were reported in ICRP Publication 110, and the specific absorbed fraction (SAF) values are presented in ICRP Publication 133. IDAC-Dose 2.1 and OLINDA/EXM version 2.0 use radiation spectra obtained with the Medical Internal Radiation Dose (MIRD) program [22]. However, the two programs have differences in calculable absorbed doses in organs and tissues. The range of alpha-particles is short, and the SAF in organs is strongly affected by the absorbed dose calculation.

The aim of this study was to calculate and compare the absorbed doses of free ^211^At and ^211^At-MABG in various organs in normal mice using two software programs, OLINDA/EXM and IDAC-Dose 2.1.

## Materials and methods

### Production of ^211^At and radiosynthesis of ^211^At-MABG

^211^At was produced through the ^209^Bi(α,2n)^211^At reaction using a Sumitomo multipurpose cyclotron (MP-30, Sumitomo Heavy Industries, Ltd. Japan) in the Advanced Clinical Research Center at Fukushima Medical University, Japan. A 30 MeV alpha-particle beam was degraded to 26.5 ± 0.9 MeV by inserting 70 μm of aluminum foil to prevent the production of ^210^At. The degraded beam was used to bombard the bismuth (99.999%, Goodfellow Cambridge Ltd., Huntingdon, England) layer on an aluminum backing for 110 min with 10.9 eμA. ^211^At was isolated from the irradiated target using the dry distillation procedure reported by Lindegren et al. [23] with slight modifications. Briefly, the target was inserted in a quartz tube placed in a tube furnace preheated to 700 °C. Under helium gas flow in a 700 °C oven, vaporized ^211^At was transported from the quartz tube to an externally connected PTFE tube that was immersed in dry ice/ethanol bath. The cooled ^211^At was trapped in the PTFE tube, eluted, and recovered using 0.5 mL of chloroform. The radioactivity of ^211^At was measured using a dose calibrator (CRC-25R, Capintec Inc., Ramsey, NJ, USA), which was previously calibrated by measuring a highly radioactive ^211^At source using a Ge detector (GEM30-70, ORTEC, Oak Ridge, TN, USA) and a dose calibrator. For the quantification of ^211^At radioactivity on a Ge detector, we selected 687.0 keV (gamma-ray intensity: *Ir* = 0.261%) of gamma-ray from ^211^At, and 569.65 keV (0.311%, against the decay of ^211^At) and 897.8 keV (0.321%, against the decay of ^211^At) from the daughter nuclide ^211^Po. Gamma-ray spectrometry was also performed using a Ge detector to assign the radionuclides produced in the target and the recovery solution of ^211^At. The radioactivity of ^211^At was 263.3 MBq at the end of the bombardment, and the radiochemical purity of ^211^At was more than 99.9% at the end of recovery; there was no contamination of ^210^At. Chloroform, the recovery solvent of ^211^At, was added to 50 μL of 0.1 M NaOH aqueous solution and then removed with nitrogen gas. The remaining ^211^At was redissolved in 3 mL of saline and administered to mice. In this study, ^211^At refers to “free astatine,” which likely consists not only of ^211^At^-^, but also, to some extent, other oxidation states [24].

^211^At-MABG was prepared in accordance with a slightly modified previously published method [25, 26]. Namely, ^211^At in chloroform and *meta*-trimethylsilylbenzylguanidine hemisulfate with *N*-chlorosuccinimide were dissolved in trifluoroacetic acid and heated at 70 °C for 10 min. Crude ^211^At-MABG was purified with a Sep-Pak tC18 Plus Light Cartridge (Waters, Milford, MA). After washing with water, ^211^At-MABG was eluted with 5% ethanol aqueous solution, providing a radiochemical yield of 36.3% (decay corrected). The eluate was diluted with saline, and sodium ascorbate was added at a final concentration of 2.5%. The radiochemical purity of ^211^At-MABG was determined using reverse-phase radio-high-performance liquid chromatography (radio-HPLC), and the value was > 98%.

### Biodistribution study

The experimental procedures and care of animals were carried out with the approval of the Fukushima Medical University Institute of Animal Care and Use Committee. Normal male mice (C57BL/6 N, 9 weeks old) were administered 0.13 MBq of free ^211^At or 0.20 MBq of ^211^At-MABG by tail-vein injection. The mice were sacrificed at 5 min, and at 1, 3, 6, and 24 h after each tracer injection (*n* = 5 in each group). The radioactivities in organs (muscle, heart, lung, spleen, pancreas, white adipocyte, testis, stomach, small intestine inclusive of contents, large intestine inclusive of contents, kidneys, adrenal glands, liver, brown adipocyte, salivary gland, thyroid gland, bone, and brain) and blood were measured using a γ-counter (Wizard2®, Perkin Elmer, MA, USA). The activities in the abovementioned organs and blood were determined as the percentage of injected activity per mass (%IA/g), whereas that in the thyroid gland was determined as %IA because the gland could not be weighed accurately.

### Radiation absorbed dose calculations

The data of biodistribution in the mice were used to estimate not only the mouse radiation absorbed doses but also the human radiation absorbed doses for both free ^211^At and ^211^At-MABG. The mean radioactivity in mouse organs at 5 min, and at 1, 3, 6, and 24 h (*n* = 5 in each group) was used to calculate the time-integrated activity coefficient (Bq-h/Bq) for each organ. The calculated %IA/organ in mouse and human organs was fitted with an exponential function and integrated to obtain the number of disintegrations (time-integrated activity coefficient) for source organs using the OLINDA/EXM version 1.1 software. One to three exponential terms can be selected for the modeling process [19]. An indirect blood-based method using patient-based red marrow-to-blood ratio (RMBLR) and bone marrow mass was used to determine bone marrow self-dose. The red marrow cumulated activity (A_RM_) is generally determined using the following equation (1) [27, 28]:

1 $$ {A}_{RM}=\left[{A}_{blood}\right]\times RMBLR\times {m}_{RM- phantom}, $$

where [A_blood_] is the blood cumulated activity concentration obtained from serial whole-blood sampling and analysis of the resulting blood activity concentration-time curve, and m_RM-phantom_ is the red marrow mass (kg) of the male human phantom. The RMBLR is a correction factor representing the marrow-to-blood activity concentration ratio. The RMBLR reported by previous studies was 0.36 [27, 28].

Using the percent kg/g method [29] with mass extrapolation of 73.0 kg (ICRP 89 adult male phantom), we extrapolated data to human dosimetry. In this method, the human %ID/organ is calculated using the following equation (2) [29, 30]:

2 $$ {\left(\% ID\right)}_{human}=\left[{\left(\frac{\% ID}{m_{organ}}\right)}_{animal}\times {\left({m}_{TB}\right)}_{animal}\right]\times {\left(\frac{m_{organ}}{m_{TB}}\right)}_{human}, $$

where *m*_organ_ is the organ mass and *m*_TB_ is the total body mass. The human body mass and organ masses were taken from OLINDA/EXM version 2.0 for adult male phantoms. The mouse body mass and organ masses were measured. For the thyroid, a mass of 14 mg for a 25 g mouse model installed in OLINDA/EXM version 2.0 was used for calculations because the gland could not be weighed accurately by the kg/g method. The 25 g mouse model is the closest to the average body mass (23.3 ± 1.2 g) of the mice used in this experiment.

OLINDA/EXM version 2.0 and IDAC-Dose 2.1 were used for human absorbed dose calculations. For animal absorbed dose assessment, only OLINDA/EXM version 2.0 was used. The absorbed dose contribution from the daughter nuclides can also be included in the absorbed dose calculations.

## Results

### Biodistribution study

**Fig. 1:**
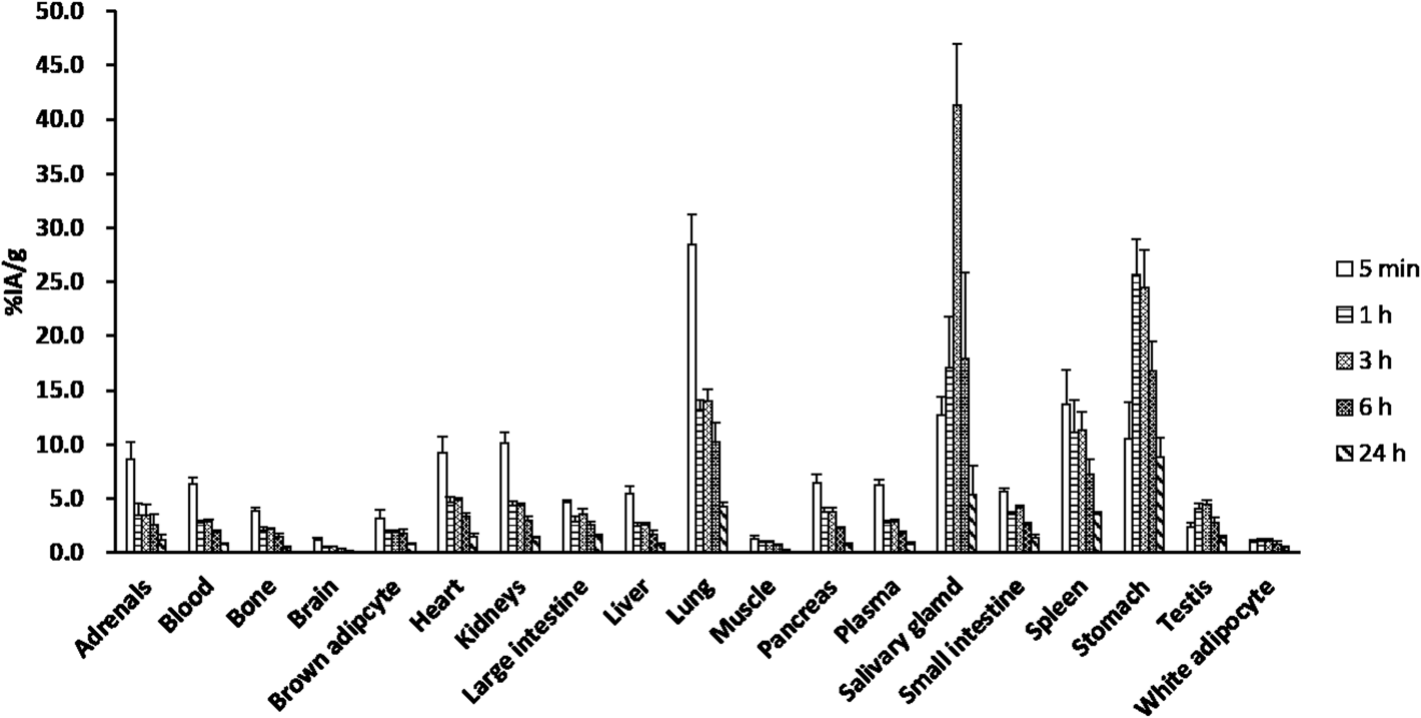
Biodistribution data of free ^211^At in mice at 5 min, and at 1, 3, 6, and 24 h. Results are shown as %IA/g (mean ± SD)

**Fig. 2:**
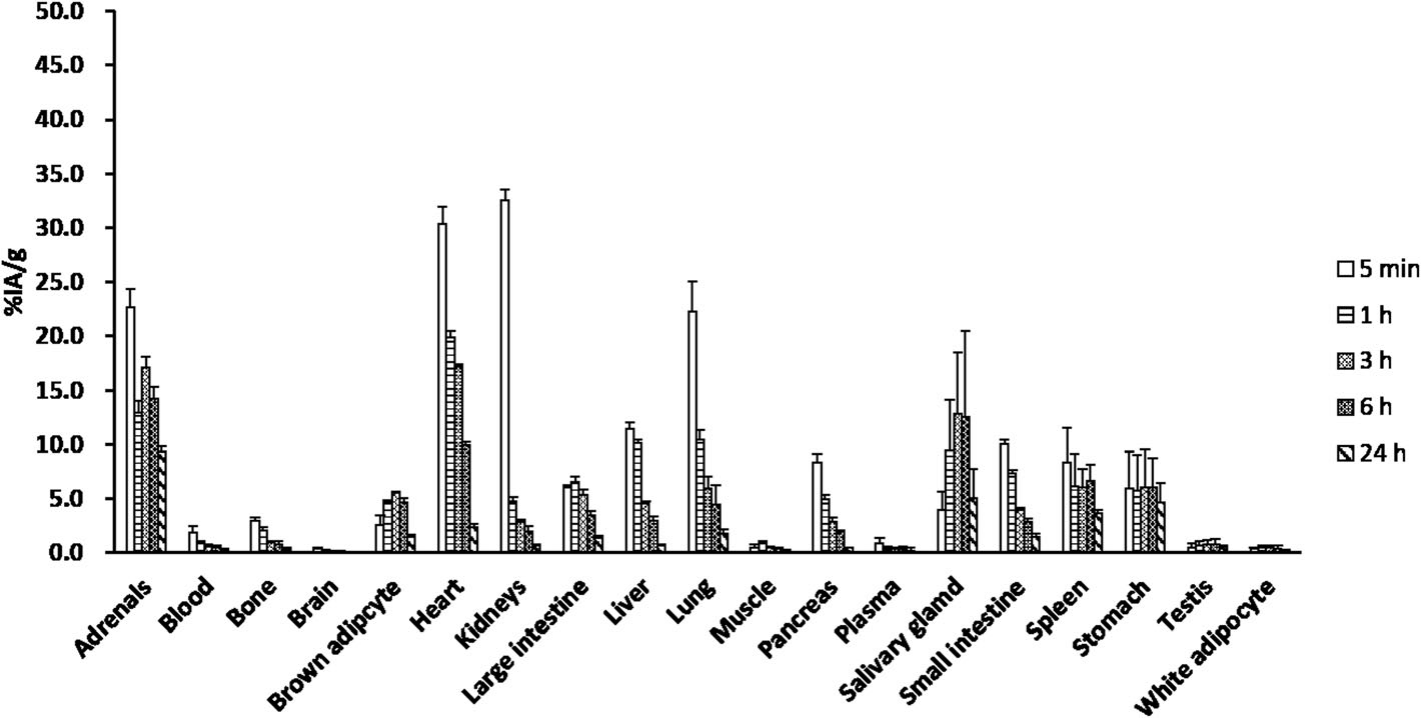
Biodistribution data of ^211^At-MABG in mice at 5 min, and at 1, 3, 6, and 24 h. Results are shown as %IA/g (mean ± SD)

The biodistributions of free ^211^At and ^211^At-MABG are shown in Figs. 1 and 2. The activity concentration of free ^211^At was higher than that of ^211^At-MABG in several organs, such as the stomach, lungs, spleen, and salivary gland. The stomach had a high concentration of free ^211^At for up to 24 h, with the highest activity concentration observed after 1 h.

On the other hand, the accumulation of ^211^At-MABG was higher than that of free ^211^At in the heart and adrenal glands. The biodistributions of the two compounds at 6 h post intravenous injection were specifically different in the heart (3.34 ± 0.33 vs. 9.92 ± 1.33%IA/g) and adrenal gland (2.52 ± 1.06 vs. 14.24 ± 2.59%IA/g). ^211^At-MABG showed faster clearance in each organ as well as in blood and plasma.

**Fig. 3:**
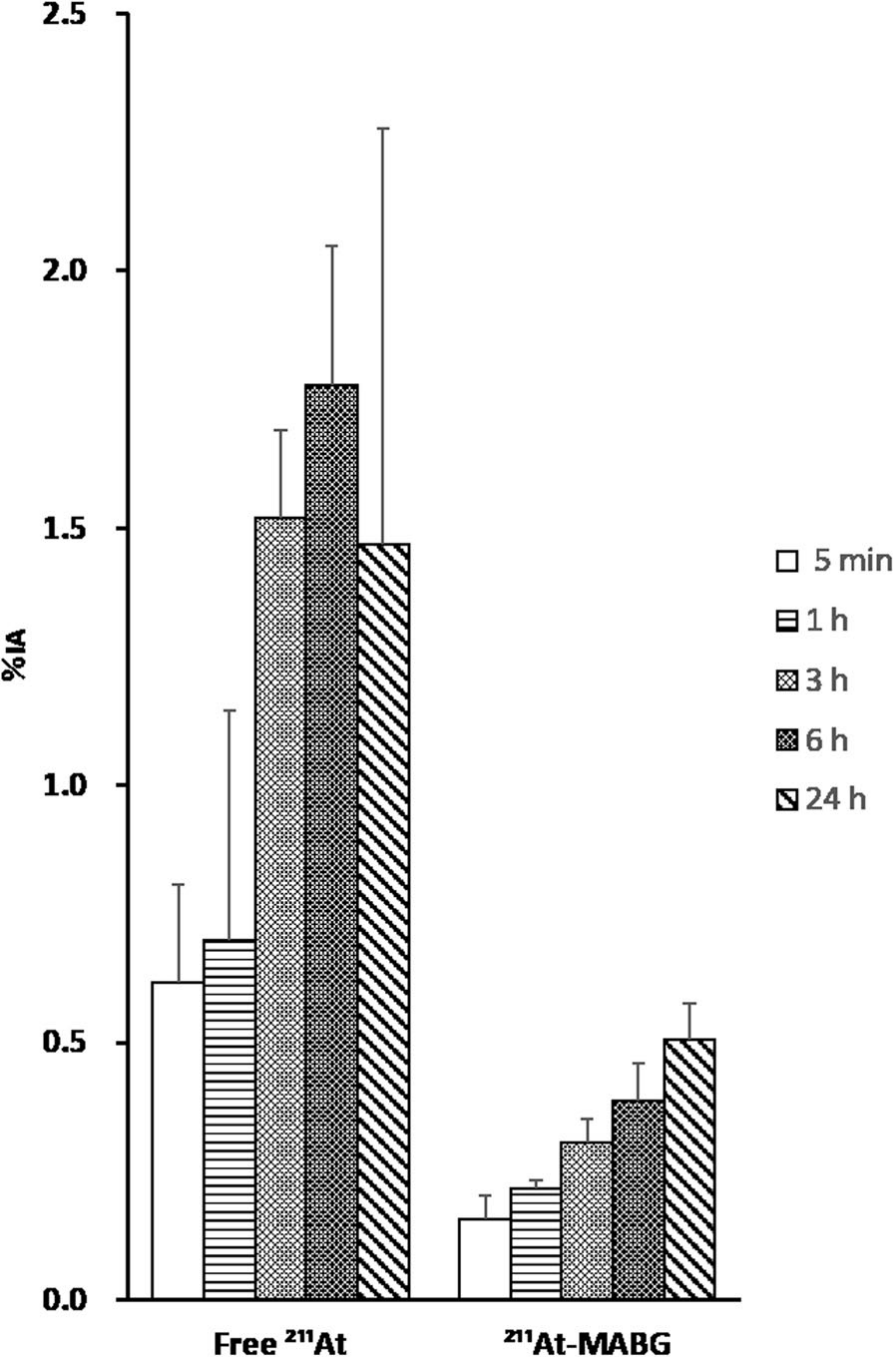
Thyroid gland biodistribution data of free ^211^At and ^211^At-MABG. Results are shown as %IA (mean ± SD)

The thyroid gland showed the highest accumulation at 6 h after the injection of free ^211^At with 1.777%IA and the highest accumulation at 24 h for ^211^At-MABG with an uptake of 0.506%IA (Fig. 3). The free ^211^At concentration in the thyroid gland was markedly increased compared with ^211^At-MABG concentration (four times higher).

### Dosimetry

The time-integrated activity coefficients calculated using OLINDA/EXM version 1.1 for the organs are listed in Table 1 for the 25 g mouse model in OLIDA/EXM version 2.0, in Table 2 for the adult male model in OLINDA/EXM version 2.0, and in Table 3 for the adult male model in IDAC-Dose 2.1. The time-integrated activity coefficients in Tables 2 and 3 were calculated by extrapolation from the distribution data from mice. The mean absorbed doses per unit injection activity for the 25 g mouse phantom estimated using free ^211^At and ^211^At-MABG biodistribution data are given in Table 4. The dosimetric calculations for free ^211^At and ^211^At-MABG showed that the thyroid received the highest absorbed dose per injection activity, with free ^211^At = 15.1 Gy/MBq and ^211^At-MABG = 4.08 Gy/MBq followed by the stomach wall in the mouse model (Table 4). Relatively higher absorbed doses in extrathyroidal tissues and organs were found in the heart, lung, and stomach wall for free ^211^At than for ^211^At-MABG.

**Table 1:** Time-integrated activity coefficient (Bq-h/Bq) for 25 g mouse model calculated using OLINDA/EXM version 1.1 for free ^211^At and ^211^At-MABG in the main target organs. Data are based on biodistribution in mouse organs

Source organ	^211^At-Free	^211^At-MABG
Brain	1.17E−02	3.90E−03
LLI* contents	5.93E−02	7.99E−02
Small intestine	2.92E−01	3.42E−01
Stomach contents	2.02E−01	5.89E−02
Heart contents	2.03E−02	5.50E−03
Kidney	1.08E−01	9.67E−02
Liver	1.83E−01	3.81E−01
Lung	1.15E−01	5.99E−02
Pancreas	3.05E−02	2.90E−02
Cortical bone	3.24E−01	2.02E−01
Spleen	5.02E−02	3.12E−02
Testis	4.12E−02	1.18E−02
Thyroid	1.49E−01	4.02E−02
Total body	1.56E+00	1.08E+00

*LLI low large intestine

**Table 2:** Time-integrated activity coefficient (Bq-h/Bq) for input to OLINDA/EXM version 2.0 for adult male model calculated using OLINDA/EXM version 1.1 for free ^211^At and ^211^At-MABG in the main target organs. It was calculated by extrapolation from the distribution data of mice

Source organ	^211^At-Free	^211^At-MABG
Adrenal gland	1.17E−03	6.13E−03
Brain	1.67E−02	5.85E−03
Left colon	1.20E−02	1.77E−02
Small intestine	8.72E−02	1.12E−01
Stomach contents	8.66E−02	2.75E−02
Right colon	1.20E−02	1.77E−02
Heart contents	3.23E−02	8.88E−03
Heart wall	3.62E−02	1.17E−01
Kidney	3.09E−02	3.14E−02
Liver	1.03E−01	2.13E−01
Lung	3.86E−01	2.11E−01
Pancreas	1.09E−02	1.04E−02
Salivary gland	4.64E−02	2.69E−02
Red marrow	2.56E−02	6.97E−03
Cortical bone	2.03E−01	1.31E−01
Trabecular bone	5.08E−02	3.27E−02
Spleen	3.69E−02	2.73E−02
Testis	6.04E−03	1.70E−03
Thyroid	6.69E−02	1.83E−02
Total body	1.49E+00	1.03E+00

**Table 3:** Time-integrated activity coefficient (Bq-h/Bq) for input to IDAC-Dose 2.1 for adult male model calculated using OLINDA/EXM version 1.1 for free ^211^At and ^211^At-MABG in the main target organs. It was calculated by extrapolation from the distribution data of mice

Source organ	^211^At-Free	^211^At-MABG
Adipose	1.49E+00	1.03E+00
Adrenal gland	1.17E−03	6.13E−03
Brain	1.67E−02	5.85E−03
Blood	3.57E−01	9.92E−02
Cortical bone mineral, volume	2.03E−01	1.31E−01
Heart wall	3.62E−02	1.17E−01
Kidney	3.09E−02	3.14E−02
Left colon wall	1.20E−02	1.77E−02
Liver	1.03E−01	2.13E−01
Lung	3.86E−01	2.11E−01
Pancreas	1.09E−02	1.04E−02
Red marrow	2.56E−02	6.97E−03
Right colon wall	1.20E−02	1.77E−02
Salivary gland	4.64E−02	2.69E−02
Small intestine wall	8.72E−02	1.12E−01
Spleen	3.69E−02	2.73E−02
Stomach wall	8.66E−02	2.75E−02
Testis	6.04E−03	1.70E−03
Thyroid	6.69E−02	1.83E−02
Trabecular bone mineral, volume	5.08E−02	3.27E−02

**Table 4:** Radiation dosimetry estimates per unit administered activity [Gy/MBq] for free ^211^At and ^211^At-MABG for 25 g mouse phantom in OLINDA/EXM ver. 2.0, based on mouse biodistribution data

Target organ	^211^At-Free	^211^At-MABG
Brain	0.037	0.012
Large intestine	0.240	0.263
Small intestine	0.336	0.348
Stomach wall	5.370	1.600
Heart	0.218	0.099
Kidney	0.518	0.463
Liver	0.153	0.317
Lung	1.910	0.994
Pancreas	0.145	0.138
Skeleton	0.094	0.065
Spleen	0.652	0.405
Testis	0.372	0.107
Thyroid	15.100	4.080
Urinary bladder	0.094	0.065
Total body	0.189	0.145

For the adult male human model, the mean absorbed doses per unit injection activity calculated using OLINDA/EXM version 2.0 and IDAC-Dose 2.1 were equivalent in major high-uptake organs such as the adrenal gland (634 vs. 517 μGy/MBq), heart (526 vs. 443 μGy/MBq), and salivary gland (458 vs. 438 μGy/MBq) (Tables 5 and 6). On the other hand, the absorbed doses in the left and right colon (21.4 vs. 134 μGy/MBq), small intestine wall (21.7 vs. 195 μGy/MBq), and stomach wall (21.3 vs. 211 Gy/MBq) calculated using OLINDA/EXM version 2.0 were markedly lower than those calculated using IDAC-Dose 2.1.

**Table 5:** Radiation dosimetry estimates per unit administered activity [μGy/MBq] from OLINDA/EXM ver. 2.0 for free ^211^At and ^211^At-MABG for human adult male phantom. It was calculated by extrapolation from the distribution data of mice

Target organ	^211^At-Free	^211^At-MABG
Adrenal gland	122.00	634.00
Brain	16.80	5.94
Esophagus	30.40	21.00
Eyes	29.70	20.60
Gallbladder wall	30.00	21.10
Left colon	30.40	21.40
Small intestine	30.60	21.70
Stomach wall	31.40	21.30
Right colon	30.10	21.10
Rectum	29.90	20.70
Heart	205.00	526.00
Kidney	145.00	147.00
Liver	83.40	172.00
Lung	466.00	255.00
Pancreas	113.00	108.00
Prostate	29.80	20.60
Salivary gland	790.00	458.00
Red marrow	103.00	59.00
Skeleton	1140.00	741.00
Spleen	357.00	264.00
Testis	250.00	70.40
Thymus	30.10	20.90
Thyroid	4840.00	1320.00
Urinary bladder	29.80	20.60
Total body	50.20	37.30

**Table 6:** Radiation dosimetry estimates per unit administered activity [μGy/MBq] from IDAC-dose 2.1 for free ^211^At and ^211^At-MABG for human adult male phantom. It was calculated by extrapolation from the distribution data of mice

Target organs	^211^At-Free	^211^At-MABG
Adrenal gland	116.00	517.00
Brain	20.20	6.80
Esophagus	21.90	6.50
Eye lenses	0.14	0.08
Gallbladder wall	4.79	1.94
Left colon wall	110.00	135.00
Small intestine wall	170.00	195.00
Stomach wall	667.00	211.00
Right colon wall	110.00	135.00
Recto-sigmoid colon wall	23.40	6.68
Heart wall	150.00	443.00
Kidney	131.00	115.00
Liver	85.70	137.00
Lung	31.10	9.24
Pancreas	110.00	92.40
Prostate	4.47	1.38
Salivary gland	758.00	438.00
Red (active) bone marrow	32.80	9.68
Endosteum (bone surface)	22.80	9.91
Spleen	266.00	182.00
Testis	241.00	67.80
Thymus	4.84	1.59
Thyroid	4160.00	1140.00
Urinary bladder wall	2.42	0.85

## Discussion

In this study, we investigated the biodistribution of free ^211^At in normal mice and compared it with that of ^211^At-MABG. We estimated the human internal radiation absorbed doses using biodistribution data from normal mice. High uptake of free ^211^At was observed in the lung, spleen, salivary gland, stomach, and thyroid, whereas ^211^At-MABG was observed in the heart and adrenal gland. In normal tissues, relatively high concentrations of ^211^At were found in the heart and adrenal gland. The low radioactivity and low retention of ^211^At-MABG in the organs where free ^211^At accumulates (stomach, spleen, salivary gland, etc.) suggest that ^211^At-MABG was relatively stable and did not undergo ^211^At deastatination in the body. Absorbed dose calculations showed that the mean absorbed dose of ^211^At was highest in the thyroid gland.

Note that the biodistribution of free ^211^At showed high free ^211^At activity concentrations in the lung and spleen. This supports the findings of a previous study where the uptake of free ^211^At was high in the thyroid gland, lung, spleen, and stomach in nude mice [31]. Previous studies also demonstrated higher activity concentrations of free ^211^At than of radioiodine in extrathyroidal organs and tissues, suggesting that the uptake/transport of free ^211^At is dependent on mechanisms other than the sodium iodide symporter (NIS) [3, 24, 31, 32].

Concerning the physical properties of ^211^At [33], the energy released from electrons and photons per decay was negligible compared with that from alpha-particles. However, preclinical results should be translated to humans with caution, as the photon contribution may be greater in clinical situations.

Results from the absorbed dose calculations for the animal model showed that the thyroid received the maximum mean absorbed dose per unit injected activity of both tracers. This was expected owing to the biodistribution pattern. The mean absorbed dose in the thyroid was higher for free ^211^At than for ^211^At-MABG, which is explained by the much higher uptake of free ^211^At than of ^211^At-MABG. In the group of mice injected with ^211^At-MABG, ^211^At accumulation in the thyroid gland increased over time. This is possibly due to the deastatination of ^211^At-MABG. The adrenal gland, heart, thyroid gland, and salivary gland seem to be potential absorbed dose-limiting organs for ^211^At-MABG. In clinical settings, the tissue accumulation of ^211^At may be blocked, which would potentially reduce the mean absorbed doses in extrathyroidal tissues [3].

The uptake of ^211^At-MABG was higher in the heart and adrenal gland, which have higher densities of NET than in other organs and glands. The biodistribution data of ^211^At-MABG were consistent with those reported in previous studies [4, 5].

The absorbed dose in human have been calculated for two different tracers with radiopharmaceuticals using OLINDA/EXM version 2.0 and IDCA-Dose 2.1. Absorbed doses were calculated using the animal biodistribution data for free ^211^At and ^211^At-MABG, and calculations with IDAC-Dose 2.1 were validated using OLINDA/EXM version 2.0 with identical results in major organs. Calculations of the two programs were based on the same computational framework, so that identical radiation exposures give the same absorbed doses independently of the situation for which they are estimated. On the other hand, the absorbed doses in the left and right colon, small intestine wall, and stomach wall calculated using OLINDA/EXM version 2.0 were quite different and markably lower than those calculated using IDAC-Dose 2.1. Although the absorbed doses in these organs were relatively low, attention should be paid to the discrepancy. IDAC-Dose 2.1 can be used to calculate the absorbed doses in 47 different organs and tissues. Therefore, it can be divided into smaller parts and calculated.

The relative biological effectiveness (RBE) of alpha-particle radiation has already been discussed in reports from the US Department of Energy [34] and the MIRD Committee [35]. However, uncertainties have also been reported for the RBE of the alpha-particle radiation [9]. Therefore, in this study, the weight factor of the alpha-particle radiation is 1 and the RBE is not considered.

It is important to consider whether a similar dosimetry can be obtained even in human studies. There are also many reports in which the results were extrapolated to human dosimetry by the kg/g method with reference to the results of animal experiments [36–38]. The kg/g method is discussed in a previous report [29]. Lee et al. estimated the human-equivalent internal radiation absorbed doses of ^124^I-MIBG using PET/CT data in a murine xenograft model [38]. In their experiment, they showed that preclinical ^124^I-MIBG data can predict reasonably precise radiation dose estimates relevant to clinical situations. The results suggest the relevance of our study in mice to human dosimetry. Further studies are needed to clarify whether our results are general.

## Conclusions

^211^At-MABG is a promising radiopharmaceutical for the treatment of malignant pheochromocytoma. The distribution of ^211^At-MABG showed different uptakes in several organs compared with free ^211^At. It is suggested that ^211^At-MABG was relatively stable and did not undergo ^211^At deastatination in the body. The higher mean absorbed doses of ^211^At-MABG in the heart and adrenal glands, which have higher NET densities than in other organs and glands, was reasonable to characterize the radiopharmaceutical. Note that free ^211^At has higher mean absorbed doses in the thyroid, salivary gland, stomach, lung, and spleen than ^211^At-MABG. This finding may contribute to the understanding of the instability of ^211^At-labeled compounds in the body and at-risk organs. Some tissues is analyzed using IDAC-Dose 2.1, and OLINDA/EXM version 2.0 show differences in alpha-particle dosimetry. The characteristics of each program should be understood and taken into consideration when they are used.

## Supplementary information

**Additional file 1:** **Table S1.** Biodistribution of free ^211^At and ^211^At-MABG in normal mouse.

## Data Availability

The datasets supporting the conclusions of this article are included within the article.
