# Supplementary material for: Human dosimetry of free 211At and meta-[211At]astatobenzylguanidine (211At-MABG) estimated using preclinical biodistribution from normal mice
Source: EJNMMI Phys. 2020 Sep 22;7:58. doi: 10.1186/s40658-020-00326-7 (PMC7509022; doi:10.1186/s40658-020-00326-7)
Supplement: Supplementary file 1 — Additional file 1: Table S1. Biodistribution of free 211At and 211At-MABG in normal mouse. [file 40658_2020_326_MOESM1_ESM.docx]

**Supplementary Material**

| Supplement Table 1 Biodistribution of free ^211^At and ^211^At-MABG in normal mouse. | | | | | | | | | | | |
| --- | --- | --- | --- | --- | --- | --- | --- | --- | --- | --- | --- |
|  | ^211^At-Free (%IA/g)^*^ | | | | |  | ^211^At-MABG (%IA/g)^*^ | | | | |
|  | 5 min | 1 h | 3 h | 6 h | 24 h |  | 5 min | 1 h | 3 h | 6 h | 24 h |
| Adrenals | 8.61 ± 1.68 | 3.51 ± 1.09 | 3.47 ± 0.98 | 2.53 ± 1.06 | 1.13 ± 0.54 |  | 22.7 ± 2.65 | 12.9 ± 1.83 | 17.15 ± 2.84 | 14.24 ± 2.59 | 9.31 ± 1.32 |
| Blood | 6.33 ± 0.6 | 2.8 ± 0.19 | 2.88 ± 0.2 | 1.89 ± 0.23 | 0.78 ± 0.1 |  | 1.84 ± 0.35 | 0.88 ± 0.13 | 0.58 ± 0.07 | 0.48 ± 0.06 | 0.27 ± 0.06 |
| Bone | 3.87 ± 0.33 | 2.06 ± 0.34 | 2.16 ± 0.15 | 1.47 ± 0.29 | 0.51 ± 0.12 |  | 2.98 ± 0.46 | 2.05 ± 0.68 | 0.98 ± 0.1 | 0.78 ± 0.19 | 0.32 ± 0.07 |
| Brain | 1.2 ± 0.13 | 0.53 ± 0.06 | 0.54 ± 0.06 | 0.35 ± 0.06 | 0.13 ± 0.02 |  | 0.35 ± 0.05 | 0.19 ± 0.02 | 0.16 ± 0.02 | 0.13 ± 0.02 | 0.06 ± 0.02 |
| Brown adipcyte | 3.14 ± 0.88 | 1.89 ± 0.21 | 1.9 ± 0.18 | 1.76 ± 0.37 | 0.75 ± 0.17 |  | 2.58 ± 0.98 | 4.7 ± 0.74 | 5.53 ± 2.02 | 4.68 ± 0.45 | 1.51 ± 0.47 |
| Heart | 9.2 ± 1.55 | 4.62 ± 0.55 | 4.82 ± 0.24 | 3.34 ± 0.34 | 1.52 ± 0.25 |  | 30.4 ± 5.69 | 19.94 ± 2.94 | 17.18 ± 2.27 | 9.93 ± 1.33 | 2.4 ± 0.28 |
| Kidneys | 10.16 ± 1 | 4.35 ± 0.4 | 4.36 ± 0.25 | 2.92 ± 0.49 | 1.36 ± 0.1 |  | 32.58 ± 14.57 | 4.8 ± 0.98 | 2.85 ± 0.41 | 1.97 ± 0.3 | 0.7 ± 0.09 |
| Large intestine | 4.66 ± 0.23 | 2.85 ± 0.5 | 3.57 ± 0.48 | 2.53 ± 0.35 | 1.44 ± 0.21 |  | 6.07 ± 1.3 | 6.59 ± 0.75 | 5.36 ± 0.51 | 3.5 ± 0.29 | 1.39 ± 0.27 |
| Liver | 5.5 ± 0.66 | 2.51 ± 0.24 | 2.6 ± 0.17 | 1.7 ± 0.41 | 0.78 ± 0.06 |  | 11.42 ± 1.21 | 10.18 ± 1.1 | 4.58 ± 0.28 | 2.99 ± 1.52 | 0.7 ± 0.12 |
| Lung | 28.47 ± 2.83 | 13.22 ± 0.86 | 14.05 ± 1.11 | 10.27 ± 1.73 | 4.25 ± 0.4 |  | 22.25 ± 5.08 | 10.49 ± 2.3 | 5.99 ± 1.02 | 4.51 ± 0.5 | 1.8 ± 0.35 |
| Muscle | 1.24 ± 0.31 | 0.98 ± 0.14 | 1 ± 0.07 | 0.63 ± 0.12 | 0.23 ± 0.06 |  | 0.51 ± 0.18 | 0.97 ± 0.4 | 0.47 ± 0.1 | 0.37 ± 0.11 | 0.21 ± 0.09 |
| Pancreas | 6.44 ± 0.85 | 3.81 ± 0.37 | 3.8 ± 0.4 | 2.28 ± 0.11 | 0.78 ± 0.08 |  | 8.3 ± 1.77 | 4.97 ± 0.89 | 2.9 ± 0.52 | 1.95 ± 0.25 | 0.44 ± 0.11 |
| Plasma | 6.29 ± 0.47 | 2.78 ± 0.21 | 2.91 ± 0.15 | 1.8 ± 0.23 | 0.78 ± 0.24 |  | 0.93 ± 0.18 | 0.42 ± 0.06 | 0.39 ± 0.04 | 0.37 ± 0.07 | 0.23 ± 0.06 |
| Salivary glamd | 12.7 ± 1.75 | 17.15 ± 4.66 | 41.3 ± 5.66 | 17.91 ± 7.96 | 5.35 ± 2.72 |  | 3.96 ± 1.45 | 9.47 ± 1.36 | 12.87 ± 1.46 | 12.53 ± 1.19 | 5.08 ± 1.51 |
| Small intestine | 5.64 ± 0.36 | 3.59 ± 0.21 | 4.16 ± 0.18 | 2.52 ± 0.29 | 1.34 ± 0.29 |  | 10.04 ± 3.54 | 7.4 ± 1.07 | 3.98 ± 0.54 | 2.85 ± 0.4 | 1.45 ± 0.22 |
| Spleen | 13.7 ± 3.23 | 11.13 ± 2.96 | 11.29 ± 1.7 | 7.21 ± 1.47 | 3.57 ± 0.25 |  | 8.32 ± 2.82 | 6.19 ± 1.59 | 6.05 ± 1.58 | 6.66 ± 2.17 | 3.69 ± 0.48 |
| Stomach | 10.51 ± 3.39 | 25.71 ± 3.28 | 24.53 ± 3.48 | 16.78 ± 2.72 | 8.82 ± 1.78 |  | 5.93 ± 0.79 | 5.74 ± 1.87 | 6.07 ± 1.11 | 6.05 ± 1.1 | 4.69 ± 1.14 |
| Testis | 2.38 ± 0.4 | 4.1 ± 0.47 | 4.42 ± 0.42 | 2.79 ± 0.45 | 1.38 ± 0.17 |  | 0.47 ± 0.05 | 0.66 ± 0.04 | 0.79 ± 0.15 | 0.83 ± 0.11 | 0.54 ± 0.07 |
| Thyroid gland^#^ | 0.62 ± 0.19 | 0.70 ± 0.45 | 1.52 ± 0.17 | 1.78 ± 0.27 | 1.47 ± 0.81 |  | 0.15 ± 0.05 | 0.22 ± 0.01 | 0.31 ± 0.05 | 0.39 ± 0.07 | 0.51 ± 0.07 |
| White adipocyte | 1.02 ± 0.12 | 1.06 ± 0.19 | 1.08 ± 0.21 | 0.78 ± 0.3 | 0.51 ± 0.08 |  | 0.36 ± 0.06 | 0.48 ± 0.04 | 0.46 ± 0.09 | 0.38 ± 0.08 | 0.26 ± 0.06 |
| * Biodistribution is presented as the percentage injection activity per mass of organ | | | | | | | | | | | |
| # Thyroid grand was presented as the percentage injection activity (%IA) | | | | | | | | | | | |
|  | | | | | | | | | | | |
